# Early down-regulation of the pro-inflammatory potential of monocytes is correlated to organ dysfunction in patients after severe multiple injury: a cohort study

**DOI:** 10.1186/cc7914

**Published:** 2009-06-11

**Authors:** Chlodwig Kirchhoff, Peter Biberthaler, Wolf E Mutschler, Eugen Faist, Marianne Jochum, Siegfried Zedler

**Affiliations:** 1 Department of Orthopaedic Surgery and Traumatology, Klinikum Rechts der Isar, Technische Universitaet, Ismaningerstrasse 22, 81675 Munich, Germany; 2 Department of Orthopaedic Surgery and Traumatology, Campus Innenstadt, Ludwig-Maximilians Universitaet, Nussbaumstrasse 20, 80336 Munich, Germany; 3 Department of Surgery, Campus Grosshadern, Ludwig-Maximilians Universitaet, Munich, Germany, Marchioninistrasse 15, 81377 Munich, Germany; 4 Department of Clinical Chemistry and Clinical Biochemistry, Campus Innenstadt, Ludwig-Maximilians Universitaet, Nussbaumstrasse 20, 80336 Munich, Germany

## Abstract

**Introduction:**

Severe tissue trauma results in a general inflammatory immune response (SIRS) representing an overall inflammatory reaction of the immune system. However, there is little known about the functional alterations of monocytes in the early posttraumatic phase, characterized by the battle of the individual with the initial trauma.

**Methods:**

Thirteen patients with severe multiple injury; injury severity score (ISS) >16 points (17 to 57) were included. The cytokine synthesis profiles of monocytes were characterized on admission, and followed up 6, 12, 24, 48, and 72 hours after severe multiple injury using flow cytometry. Whole blood was challenged with lipopolysaccharide (LPS) and subsequently analyzed for intracellular monocyte-related TNF-α, IL-1β, IL-6, and IL-8. The degree of organ dysfunction was assessed using the multiple organ dysfunction syndrome (MODS)-score of Marshall on admission, 24 hours and 72 hours after injury.

**Results:**

Our data clearly show that the capacity of circulating monocytes to produce these mediators *de novo *was significantly diminished very early reaching a nadir 24 hours after severe injury followed by a rapid and nearly complete recovery another 48 hours later compared with admission and controls, respectively. In contrast to the initial injury severity, there was a significant correlation detectable between the clinical signs of multiple organ dysfunction and the *ex vivo *cytokine response.

**Conclusions:**

As our data derived from very narrow intervals of measurements, they might contribute to a more detailed understanding of the early immune alterations recognized after severe trauma. It can be concluded that indeed as previously postulated an immediate hyperactivation of circulating monocytes is rapidly followed by a substantial paralysis of cell function. Moreover, our findings clearly demonstrate that the restricted capacity of monocytes to produce proinflammatory cytokines after severe injury is not only an *in vitro *phenomenon but also undistinguishable associated with the onset of organ dysfunction in the clinical scenario.

## Introduction

The pathophysiological immune alterations following severe multiple injury are predominantly directed by products of danger-signal-triggered monocytic hyperactivation [1]. Circulating blood monocytes express a plethora of mediators, including the major proinflammatory cytokines IL-1β, IL-6, IL-8, and TNFα. These cytokines are often considered the engine driving the inflammatory response of the entire organism referred to as systemic inflammatory response syndrome [2]. As higher concentrations of pro-inflammatory cytokines are deleterious, a counter-regulatory response is initiated to dampen the inflammatory process [3-5]. Any imbalance of the tightly regulated homeostasis of pro- and anti-inflammatory forces causes either a hyperinflammatory or an immunosuppressive state [6-8]. Such dyshomeostasis often results in an uncontrollable cellular dysfunction clinically appearing as multiple organ dysfunction syndrome (MODS) [9,10]. There is growing evidence that the functional depression of monocytes in particular might contribute to infectious susceptibility and late mortality in critical illness [11]. In most current investigations, monocyte-related inflammatory activity was determined in biologic fluids from mixed or purified monocyte cultures or in whole blood challenged with lipopolysaccharide (LPS), respectively [12,13]. A crucial drawback using of bulk production assays, such as ELISA, for the determination of cytokines in supernatants is the fact that they measure accumulated proteins over the incubation time. They become impractical when large numbers of heterogeneous cell populations are to be analyzed *ex vivo *and restrictively only give global information reflecting the properties of the entire cell population being studied. In contrast, the investigation of cytokine *de novo *synthesis via multiparametric flow cytometry can provide single cell information at a specific time point, with phenotypic markers on the surface but without such a summation effect [14]. Furthermore, intracellular cytokine staining remains unaffected by the short half-life of the proinflammatory mediators or the presence of soluble cytokine inhibitors, such as sIL-1RA and sTNF-Rs, respectively.

Therefore, the purpose of this study was to monitor the capability of peripheral blood monocytes of patients with multiple injuries during the early posttraumatic course on a single cell level and to correlate the results to clinical parameters of MODS.

## Materials and methods

The study was performed at our academic level 1 trauma center according to the guidelines of Good Clinical Practice after approval by the local ethics committee (reference number 012/00). Written informed consent was obtained from each patient when the patient was conscious or if the patient was still unconscious, from the next of kin or a legal representative. Healthy laboratory and hospital employees of both genders served as a control group. Written informed consent was also obtained from each healthy volunteer.

### Patient management and treatment

Patients between 18 and 75 years of age with multiple injuries (New Injury Severity Score (NISS) of >16 points) admitted to the trauma shock unit within 90 minutes after the traumatic event were included. Patients suffering from an isolated traumatic brain injury, receiving splenectomy or deceasing within the first 48 hours of hospital stay were not included. Patients with a history of steroid use, anti-inflammatory treatment, or hormone replacement therapy were not included. Patients with malignancies or chronic diseases of the liver, kidneys, or lungs were also not accepted. Fractures were stabilized as soon as possible by definitive internal fixation or alternatively by temporary external stabilization. Every patient routinely received a second-generation cephalosporin antibiotic (cefuroxime intravenously 1500 mg morning-noon-evening (1-1-1)) either due to open injury or post-operatively.

### Clinical parameter and outcome evaluation

MODS was assessed on admission, and 24 hours and 72 hours after injury using the score by Marshall and colleagues [15]. As previously described, we assumed MODS with a score of more than 12 points on two consecutive days or at least three days during the observed period [16]. The patients' outcomes were evaluated 90 days after injury.

### Blood sampling and stimulation

Arterial blood samples (5 mL) from the patients were drawn in sterile heparinized (2500 IU) tubes, on admission, and 6, 12, 24, 48, and 72 hours after the traumatic event and processed within 30 minutes after collection. Heparinized blood samples from healthy donors were obtained once. The whole blood samples were diluted 1:10 in RPMI 1640 medium with 25 mmol/mM HEPES buffer and L-glutamine (Invitrogen, Karlsruhe, Germany) and supplemented with 10% fetal calf serum (Vitromex, Vilshofen, Germany) and 0.1 mg/mL gentamicin (Merck, Darmstadt, Germany). All reagents used to suspend the cells were LPS-free. Aliquots of 1 mL were challenged with 1 μg/mL LPS (from *Escherichia coli*, Serotype 055:B5, Sigma-Aldrich, Deisenhofen, Germany) for four hours at 37°C and 5% carbon dioxide (CO_2_) with 2 μM monensin (Sigma-Aldrich, Deisenhofen, Germany) *ab initio *to inhibit protein secretion. In parallel incubation of samples without LPS served as negative controls. After four hours of stimulation samples were washed twice, first with 500 μL, then with 1 mL PBS supplemented with 0.1% sodium azide (NaN_3_) and stained for flow cytometric analysis.

### Phenotyping and intracellular cytokine staining of peripheral monocytes

The resulting pellets were incubated with 5 μL of pre-titrated monoclonal anti CD14 antibodies (IgG2a, mouse) conjugated to phycoerythrin-cyanin-5 (PE-Cy5) (Beckman-Coulter, Krefeld, Germany) in order to identify monocytes by their surface phenotype. Cells were kept for 20 minutes on ice in the dark for fluorescent labeling and then fixed using 100 μL Intra Prep™ fixation reagent (Beckman-Coulter, Krefeld, Germany). During fixation, samples were vigorously mixed to avoid cell clumping. Then the cells were washed twice in PBS/0.1% NaN_3 _and resuspended in 300 μL PBS supplemented with 0.1% NaN_3 _and 20% human AB+ serum (Sigma-Aldrich, Deisenhofen, Germany) for 30 minutes at 4°C to reduce the possibility of nonspecific antibody binding to Fc-receptors. Once washed with 700 μL PBS/0.1% NaN_3_, pellets were treated with 100 μL Intra Prep™ permeabilization reagent (Beckman-Coulter, Krefeld, Germany) for 10 minutes at room temperature in the dark to generate gaps in the membranes. Then aliquots of 30 μL volume were stained with 1 μL of a 100 μg/mL solution of fluoresceinisothiocyanate (FITC)- or PE-labeled monoclonal antibodies specific for IL-1β, IL-6, TNFα (all IgG1, mouse, Hoelzel Diagnostika, Cologne, Germany), or IL-8 (IgG1, mouse, Biosource, Solingen, Germany) for 25 minutes at room temperature in the dark. In addition, irrelevant isotypic antibodies (BD Pharmingen, Heidelberg, Germany) were used to verify the staining specificity of the experimental antibodies. After a washing step with PBS/0.1% NaN_3_, cells were resuspended in isotonic solution (Isoton II^®^, Beckman-Coulter, Krefeld, Germany) and analyzed on a flow cytometer immediately.

### Multiparameter flow cytometric analysis

**Figure 1:**
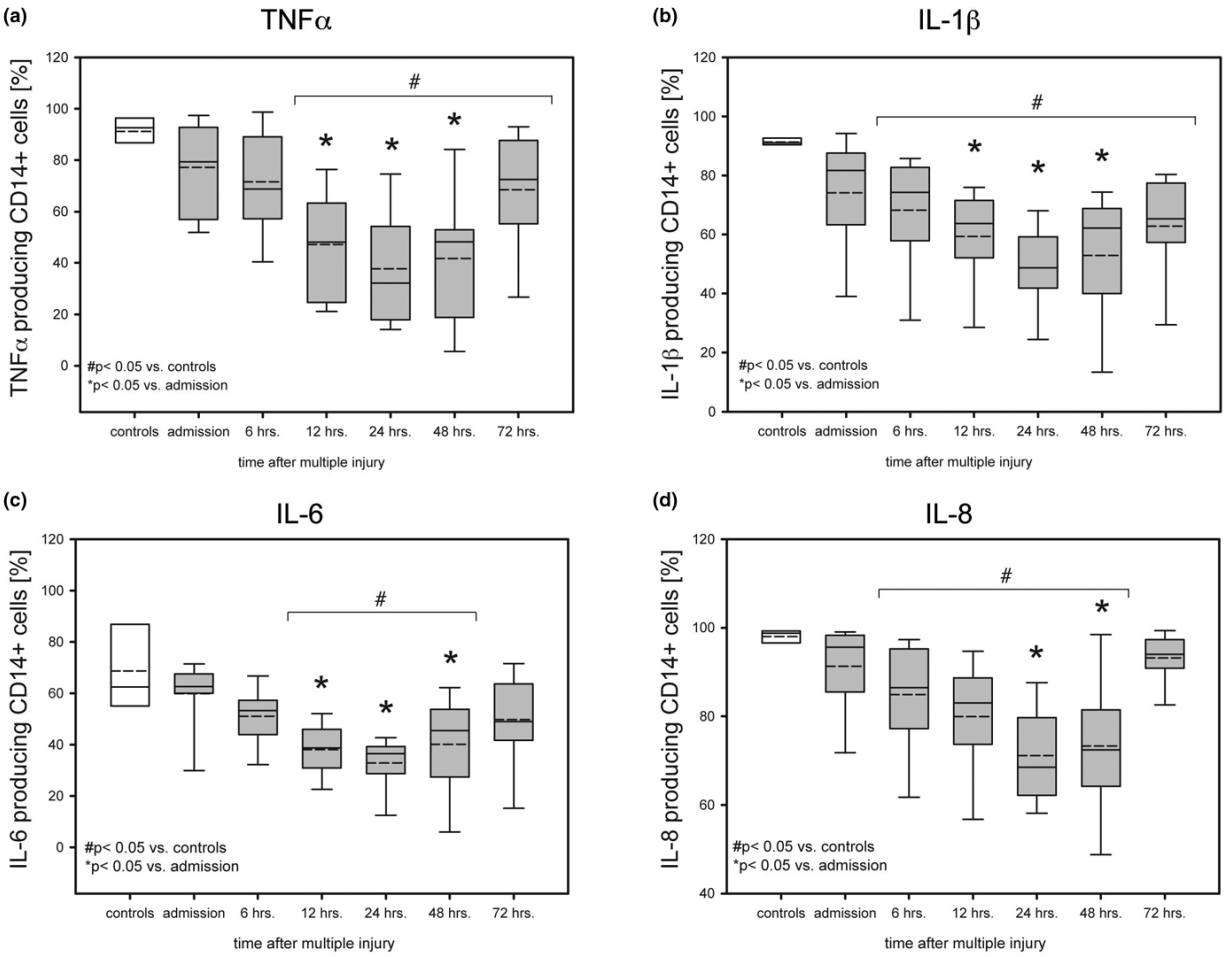
Severe multiple injury results in a rapid decline of intracellular cytokine synthesis by monocytes within the first 24 hours after trauma. Cytokine *de novo *synthesis capacity was determined using an *ex vivo *whole blood approach in response to lipopolysaccharide (LPS). Results are calculated as percentage of cytokine positive CD14+ monocytes. Blood samples were drawn on admission, 6, 12, 24, 48, and 72 hours post trauma. Data are given as boxplots (median, 5th, 95th percentile). n = 13 patients (grey), n = 8 controls (white). ^# ^*P *< 0.05 vs. control group; * *P *< 0.05 vs. admission values.

**Figure 2:**
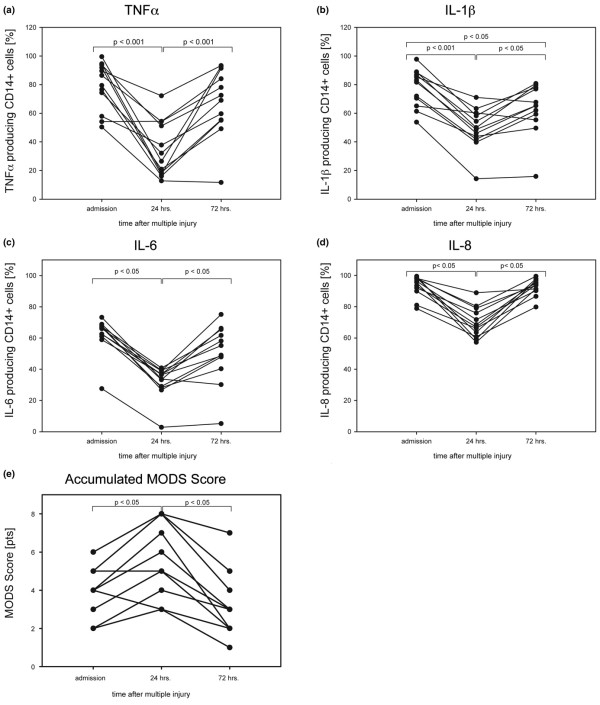
Individual time courses. **(a to d) **Results for cytokine *de novo *synthesis capacities and **(d) **the accumulated multiple organ dysfunction syndrome (MODS) score are depicted for each patient on admission, 24 hours, and 72 hours after trauma, respectively. Results are calculated as percentage of cytokine positive CD14+ monocytes. All patients showed a significant amelioration of organ function 72 hours after admission to the ICU. Data are given as boxplots (median, 5th, 95th percentile). n = 13 patients.

For flow cytometric analysis an Epics™ XL MCL flow cytometer (Beckman-Coulter, Krefeld, Germany) was used, fitted with an air-cooled 15 mW 488 nm argon ion laser, and filter settings for FITC (525 nm), PE (575 nm), and PE-Cy5 emitting in the deep red (675 nm). Data acquisition on the flow cytometer was obtained with System II™ Software (Beckman-Coulter, Krefeld, Germany). After appropriate instrument settings and spectral compensations, instrument alignment and fluidics were regularly verified using FlowCheck™ beads (Beckman-Coulter, Krefeld, Germany). A minimum of 5000 events was computed in list mode using log-amplified fluorescence signals and linearly amplified side- and forward-scatter signals. The data were analyzed using free WinMDI™ Software (Version 2.8, Bio-Soft Net [17]). A gate was set around the monocyte population, which was most strongly positive for CD14 on side scatter versus PE-Cy5 (CD14) dot plots, in order to exclude lymphocytes and debris from data analysis. Histograms representing the mean fluorescence intensity (MFI) of unstimulated cells were used as a guide for setting cutoff markers to delineate positive and negative populations. Even MFI of CD14 surface receptor expression was determined by a logarithmic scale, whereas results of cytokine *de novo *synthesis are shown as percentage of cytokine containing CD14+ monocytes after *ex vivo *stimulation with LPS (Figures 1 and 2).

### Statistics

The statistical spread of the means is principally given as the standard error of the mean. Data are plotted as boxes (interquartile range that contains the 50% of values) with a straight line at the median, a dashed line at the mean, and error bars defining the 5th and the 95th percentiles. Differences in the experimental means were considered to be significant if *P *< 0.05, as determined by repeated measures (RM) analysis of variance on ranks for non-parametric data and adequate *post hoc *procedures for multiple comparisons, when appropriate. For pairwise comparisons the student's t-test was used. Correlation analysis between MODS score and *de novo *synthesis capacity was performed using the calculation of Spearman rank order, as the patients were not normally distributed. The level of significance was set at *P *< 0.001. Statistical analysis was performed using Sigma Stat 3.1 software (Systat Inc., Chicago, IL, USA).

## Results

### Patient collective and clinical data

In this study 13 patients (four females and nine males) were enrolled with an average age of 41 ± 5 years and a mean NISS of 32 ± 3 points. MODS score accounted for 4.2 ± 0.4 points on admission, for 5.9 ± 0.5 points 24 hours after injury and for 3.2 ± 0.5 points 72 hours after injury (*P *< 0.05). In none of the patients did the MODS score exceed 10 points within the observation period (Figure 2e). However, three of the patients developed severe multiple organ failure (MOF) in the later posttraumatic course (MODS score >12 points) and one patient (patient II) died 80 days after trauma due to MOF. None of the patients included in the study yielded a positive blood culture for Gram-negative or Gram-positive microorganisms up to the sixth posttraumatic day. Clinical data concerning injury patterns, age, initial NISS, and MODS score, as well as the clinical outcome 90 days after the traumatic event are depicted in Table 1. The control group included eight healthy volunteers (four females and four males) with a mean age of 46 ± 14 years.

**Table 1:** Clinical characteristics of the patient population after multiple injury

**Patient number**	**NISS**	**MODS score** **Admission, 24 hours, 72 hours**			**Outcome after 90 days**	**Injury pattern**
I	33	4	3	2	neurological deficits upper extremity	subtotal amputation upper limb, bilateral pulmonary contusion, minor scalp laceration
II	33	3	5	2	deceased due to MOF	moderate HI, pulmonary contusion, subtotal amputation lower extremity
III	20	6	8	5	complete recovery	pulmonary contusion, blunt abdominal trauma
IV	17	2	3	1	complete recovery	minor HI, pulmonary contusion, fx upper extremity, multiple fx lower ext.
V	22	2	4	3	complete recovery	displaced trimalleolar fx, unilateral pulmonary contusion, lumbar vertebral body fx
VI	24	4	6	3	rehabilitation hospital	minor HI, pulmonary contusion, serial rib fx, pelvic fx
VII	29	5	5	2	complete recovery	bilateral pulmonary contusion, serial rib fx, sinistral femur shaft fx, III open tibia fx
VIII	57	6	8	7	neurological deficits due to HI	severe HI, pulmonary contusion, cardiac contusion, serial rib fx, cervical spine fx, liver rupture
IX	34	3	5	3	disabled by missing right upper extremity, no neurological deficits	moderate HI, pulmonary contusion, cervical spine fx, traumatic amputation in the upper extremity shoulder joint, open book pelvic fx, multiple fx lower extremities
X	36	5	8	5	disabled by significant neurological deficits due to the brain injury	severe HI, intracerebral bleeding, bilateral pulmonary contusion, sinistral serial rib fx C2-10, hemopneumothorax, spleen hematoma
XI	34	6	8	4	complete recovery	thoracic trauma, cardiac contusion, blunt abdominal trauma, liver rupture, renal contusion, cervical spine fx, bilateral fx upper extremities
XII	41	4	6	3	disabled by significant neurological deficits due to the brain injury	moderate HI, cranial fx (Le fort III), pulmonary contusion, serial rib fx, pelvic fx, multiple open fx lower extremities
XIII	38	4	7	4	physical therapy	moderate HI, cranial fx (Le fort III), lower limb fx

Multiple organ dysfunction syndrome (MODS) score was calculated on admission (adm), 24 hours, and 72 hours after injury. NISS = New Injury Severity Score; MOF = multiple organ failure; HI = head injury; fx = fracture.

### Monocyte frequencies and CD14 surface receptor expression

The differential blood count revealed unchanged relative frequencies of monocytes in the early posttraumatic course with values ranging from 5.6 ± 3.4% on admission to 6.2 ± 0.8% 72 hours after severe multiple injury. In addition, analysis of the CD14 MFI, as an indirect measurement of the surface receptor density, yielded no significant difference within the first 72 hours after trauma (78.4 ± 10.7 on admission vs. 74.7 ± 13.5 after 24 hours and 76.5 ± 13.1 after 72 hours; Table 2).

**Table 2:** The differential blood count revealed unchanged relative frequencies (%) of monocytes in the early post traumatic course

**Time post trauma**	**Admission**	**6 hours**	**12 hours**	**24 hours**	**48 hours**	**72 hours**
**Monocytes %**	5.6 ± 3.4	6.5 ± 1.1	5.7 ± 2.6	5.8 ± 0.9	5.7 ± 2.0	6.2 ± 0.8
**CD14 (MFI)**	78.4 ± 10.7	81.3 ± 11.3	79.3 ± 9.8	74.7 ± 13.5	79.8 ± 9.8	76.5 ± 13.1

Even the CD14 receptor density on the surface of monocytes, analyzed as mean fluorescence intensity (MFI) via flow cytometry showed hardly any fluctuations in patients on admission, 6, 12, 24, 48, and 72 hours after injury. Values are presented as mean +/- standard error of the mean.

### Intracellular expression of TNFα, IL-1β, IL-6, and IL-8

**Figure 3:**
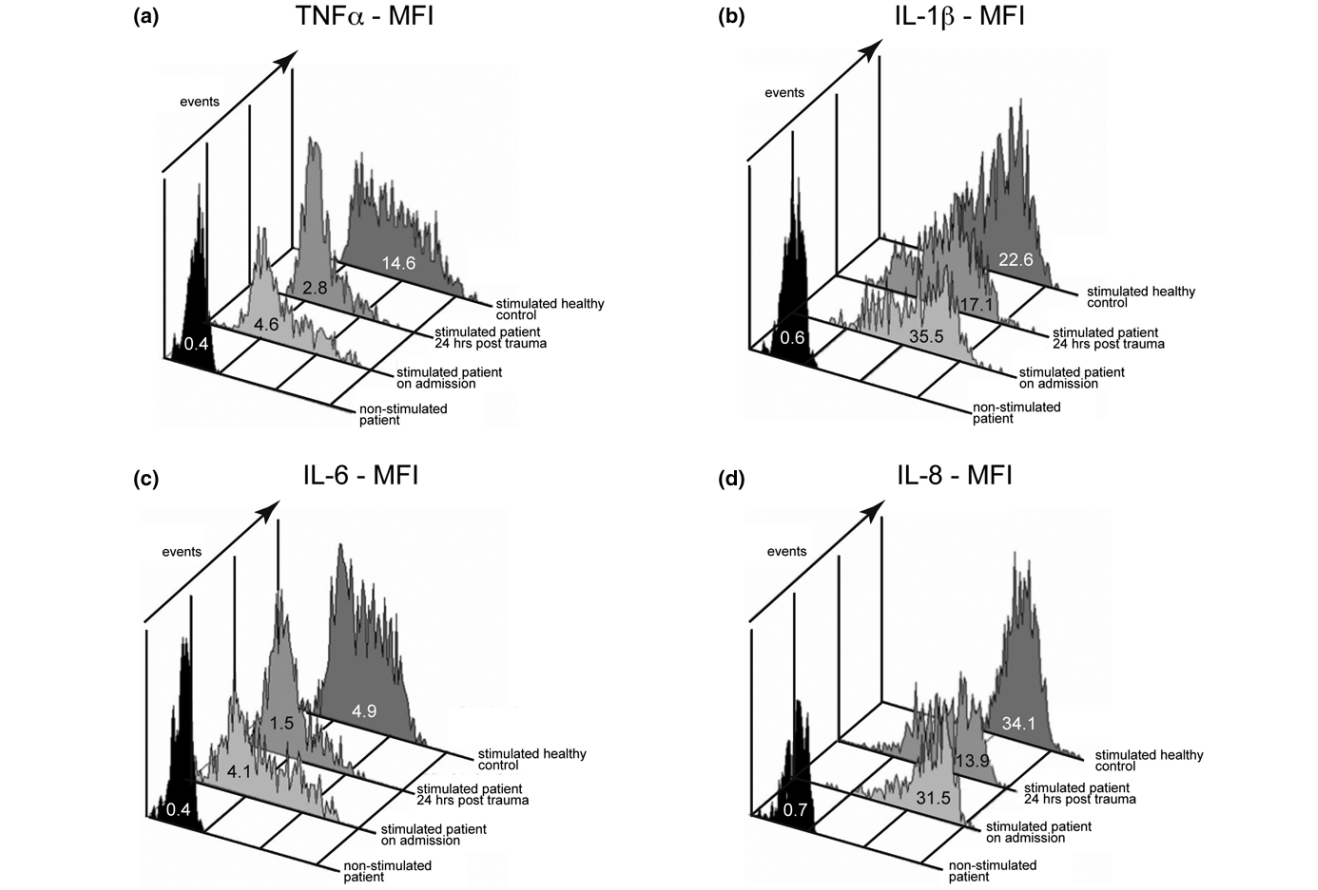
Representative fluorescence histograms displaying the internal content of IL-1β, IL-6, IL-8, and TNFα in CD14+ monocytes of one patient on admission, and 24 hours post trauma vs. one control. Whole blood samples were analyzed either after short-term stimulation with lipopolysaccharide or unchallenged as a guide for setting markers to delineate positive and negative cell populations. For relative quantification of the amount of synthesized cytokines the mean fluorescence intensity (MFI) was calculated.

In patients and healthy controls, significant cytokine reactivity was not detectable in unchallenged whole blood samples. However, after four hours of stimulation with LPS in the presence of monensin, the cytokine response of healthy peripheral blood monocytes was markedly upregulated yielding 91.3 ± 0.6% CD14+IL-1β +, 68.7 ± 5.6% CD14+IL-6+, 98.0 ± 0.6% CD14+IL-8+, and 91.3 ± 2.2% CD14+TNFα + cells (Figure 1). Figure 3 displays a typical set of MFI histograms representing the effect of LPS on the IL-1β, IL-6, IL-8, and TNFα *de novo *synthesis in peripheral blood monocytes of a patient with multiple injuries as compared with a healthy control. The cumulative percentage of LPS-stimulated 'traumatized' monocytes staining positive for IL-1β was already significantly diminished six hours after the traumatic impact with 68.3 ± 5.2% followed by 59.3 ± 4.4% after 12 hours, reaching its lowest levels with 48.7 ± 3.9% after 24 hours, and finally showing a very slow increase towards normal levels after 48 hours with 53.0 ± 5.8% and 62.8 ± 4.7% 72 hours post trauma compared with controls (Figure 1b). The behavior of the other mediators analyzed in the early posttraumatic course was similar. With regard to IL-8, the number of producing cells continuously declined between six hours (86.5 ± 3.4%) and 24 hours (68.5 ± 2.8%) post injury subsequently returning to normal after 72 hours (94.0 ± 1.5%; Figure 1d). Slightly different from IL-1β (88.7 to 93.8%) and IL-8 (94.6 to 99.5%), the frequencies of TNFα (79.8 to 99.2%) and in particular IL-6 (53.7 to 91.5%) synthesizing monocytes in response to LPS revealed a broader range of variation in volunteers. Consequently in patients the reduced capacities to produce both mediators did not reach statistical significance until 12 hours after trauma (TNFα: 47.2 ± 5.9%, IL-6: 38.0 ± 2.8), touching bottom after 24 hours (TNFα: 38.0 ± 6.1%; IL-6: 32.9 ± 2.9%), and slowly rising again to nearly admission values 72 hours after injury (TNFα: 68.5 ± 6.3%; IL-6: 49.7 ± 5.0; Figures 1a and 1c). Altogether the *de novo *synthesis capacity of IL-1β, IL-6, IL-8, and TNFα directly after severe injury decreased significantly in relation to controls reaching a nadir 24 hours post injury compared with the values obtained on admission (Figure 1). Most interestingly, between 24 hours and 72 hours after the impact, a functional conversion occurred in the monocyte subset with strong increasing percentages of producing cells in response to LPS towards admission values (*P *< 0.05; Figure 2).

### Comparison to clinical data

**Figure 4:**
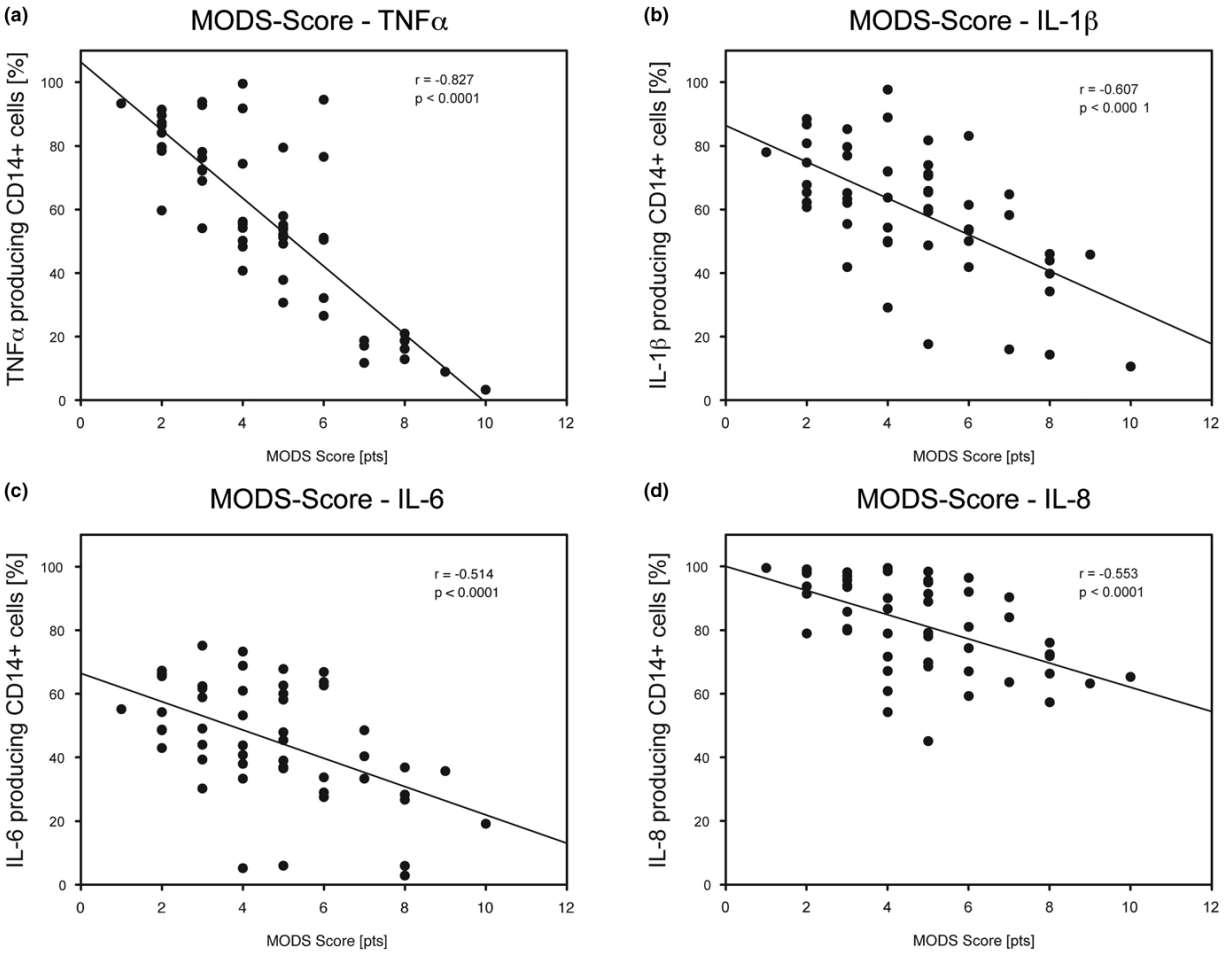
Significant correlations of cytokine *de novo *synthesis capacity of TNFα, IL-1β, IL-6, and IL-8 in monocytes with MODS score over a time period of 72 hours post trauma. **(a) **TNFα: r = -0.827, *P *< 0.0001; **(b) **IL-1β r = -0.607, *P *< 0.0001; **(c) **IL-6 r = -0.514, *P *< 0.0001; **(d) **IL-8 r = -0.553, *P *< 0.0001. The correlation coefficients were calculated using Spearman rank order, n = 13 patients. MODS = multiple organ dysfunction syndrome.

The capacity of TNFα *de novo *synthesis significantly correlated with the accumulated MODS score (r = -0.827, *P *< 0.0001). Likewise, the IL-1β capacity and MODS score showed a strong correlation (r = -0.607, *P *< 0.0001). Correlations of IL-6 and MODS score as well as IL-8 and MODS score were not that strong (r = -0.514, *P *< 0.0001; r = -0.553, *P *< 0.0001, respectively). The regression curves are depicted in Figure 4.

## Discussion

Although the impact of proinflammatory cytokines under septic conditions has been well established, its role after severe traumatic injury is not as well delineated. However, there is strong evidence suggesting that the overwhelming release of proinflammatory cytokines is one crucial initiating event in the posttraumatic acute phase response. However, a comprehensive understanding of the underlying mechanisms is still missing. In particular, the importance of the major proinflammatory cytokines IL-1β, IL-6, IL-8, and TNFα in patients with multiple injuries were investigated in many studies (for review see [18]). Patients reveal elevated systemic TNFα levels very early, within hours after the traumatic insult, then rapidly declining levels, whereas the chance to detect IL-1β is less likely to be due to its extreme short half-life [18]. Both cytokines have similar effects, and despite their brief appearance in circulation they show essential metabolic and hemodynamic effects, activating mediators downstream in the cytokine cascade such as IL-6 and IL-8. Notably, for that reason measurements of cytokine blood levels in severely ill patients do not allow precise determinations of cytokine synthesis and secretion *in vivo*. In addition, the interpretation of cytokine data is complicated by the fact that some mediators, for example IL-1β and TNFα, exist in cell-associated and systemically released forms with different biochemical properties, aggravating the exact quantification of bound proteins by assays for circulating cytokines [19]. However, the most crucial point is that the sole determination of cytokine levels in circulation is restricted to reflect the global production irrespective of the cellular origin. Thus, an adequate evaluation of the inflammatory balance is more complex than just measuring cytokine concentrations in body fluids, requiring appropriate methodical approaches. In fact, contradictory findings regarding posttraumatic cytokine plasma levels support the notion that not only the systemic levels are of special interest, but also the synthesis capacity of single peripheral blood cells.

Against this background, in this study we focused on the hyperinflammatory activity of peripheral monocytes after severe multiple injury, which starts within minutes after the traumatic impact. These important cells of the innate immunity have been suggested to be one of the major sources of proinflammatory mediators in the early posttraumatic course [20,21]. However, neither analysis of mixed cell culture supernatants nor determination of systemic cytokine levels is suited for the evaluation of the proteins' cellular origin. On the contrary, flow cytometry represents a reliable method enabling highly selective and reproducible monitoring of the functional status of specific immune cell subsets [22-24].

Applying this sophisticated technique we found a significant reduction of LPS reactivity in circulating monocytes staining positive for TNFα, IL-1β, IL-6, and IL-8 between 12 and 48 hours after injury. Besides the timely kinetics, not all cytokines behave in a similar way, albeit that none of the investigated cytokines maintained its secretion capacity. In particular, 24 hours after trauma TNFα *de novo *synthesis was strongest blunted after LPS challenge followed by IL-6, IL-1β, and finally IL-8, which was also reduced but not as much as the previous mentioned mediators. It is well documented that the initial and rather short hyperactivation of monocytes in surgical patients results in a hypo-responsive state towards restimulation *ex vivo *with inflammatory stimuli such as bacterial LPS. This immunosuppression was characterized as being either partially compensated after three to five days owing to the influx of new and immature monocytes [25] or lasting several days in trauma patients after admission [26], respectively. But it is noteworthy that alterations of monocyte reactivity are a reflection of subtle modifications that differ depending on the nature of the stress, such as major surgery, trauma, or burn [27].

Utilizing a flow cytometric approach, we provide essential evidence that patients with multiple injuries already display a significant functional impairment of monocytes within six hours after the traumatic event compared with healthy volunteers. That applies to IL-1β as well as IL-8, whereas the diminished TNFα and IL-6 synthesis lagged six hours behind probably due to bigger statistical variations in the control group. Except for IL-8, the proportions of *de novo *synthesizing monocytes were also not significantly reduced until 12 hours after admission compared with the baseline values (IL-8 after 24 hours), all finally reaching a nadir 24 hours after trauma. In the further time course, all analyzed cytokines showed a rapid and nearly complete recovery towards normal. A strong tendency towards normal was detectable, even if the continuous monitoring period was completed 72 hours after admission. In our opinion these findings reflect either the above mentioned refractory state of monocytes towards endotoxin challenge already having released cytokines after the traumatic impact and not yet replaced by their successors from the bone marrow. On the other hand, the influx of newly recruited and possibly immature monocytes may lack the full spectrum of activity as compared with their predecessors.

This study has to be taken in context to the previous work of Spolarics and colleagues [28]. They focused on the later course of patients following multiple injury assessing TNFα, IL-6, and IL-1β on day 2, 5, and 10 post injury. They reported a rate of 40% TNFα +, 50% IL-6+, and 60% IL-1β + monocytes on day 2 after trauma. This is absolutely in line with our data. However, they further observed a decrease of each cytokine on day 5 and 10 [28]. In summary, both studies might complement one another and suggest that monocytic capacity recovers up to 72 hours after trauma followed by a second decrease up to day 10 post injury. Although, this is notional and a study characterizing the early as well as the late course seems to be necessary.

Previous studies failed in correlating blood plasma levels of TNFα and IL-1β with the development of organ dysfunction [29-31]. These negative findings might be due to the already mentioned short half-life of both mediators [18]. Overcoming this biologic drawback using intracellular cytokine analysis we found a strong inverse correlation of IL-1β and TNFα synthesis capacities and the occurrence of MODS. Our data also indicated a significantly impaired monocytic IL-6 and IL-8 *de novo *production related to the clinical signs of organ dysfunction. In the present study, MODS was assessed using the widely accepted validated Marshall score, which is not free of criticism. Sauaia and colleagues recently compared the Marshall score with the Denver trauma score [32] clearly demonstrating that both scores perform reasonably well as indicators of adverse outcomes in critically ill patients. The Denver MOF score, however, performed slightly better due to greater specificity. Due to its simplicity the Denver MOF score might be a more attractive tool to be used in future clinical research both as an outcome tool in trials, and in risk adjustment as well as a monitoring device at the bedside.

It seems reasonable to assume that the intensity of monocytic temporal paralysis, that is, the diminished capacity to release proinflammatory cytokines in response to LPS, is in direct proportion to the development of early MODS in seriously injured patients, probably leading to a higher susceptibility to infections and late MODS. Several studies demonstrated that patients with MODS had higher plasma levels of IL-6 than patients without organ dysfunction [33-35] or showed correlations of increased IL-8 plasma levels with the severity of injury and the development of complications during the early post-traumatic course [36]. However, most studies assessed cytokine levels in mixed blood cell cultures disregarding the fact that other peripheral blood cells are also potentially inducible to secrete cytokines in response to the same stimulants. Furthermore, the proportion of different cell types may vary after trauma, especially from patient to patient, according to the severity of injury. In our opinion, to date the sole determination of cytokines in whole blood or in the supernatants of peripheral blood mononuclear cell cultures, respectively, does not represent an adequate method to prove possible changes in monocyte reactivity, because they do not exclusively consist of this single subset. In principle, data concerning blood cytokine levels after traumatic injury are often contradictory and their interpretation is complicated by the fact that not only peripheral blood cells contribute to systemic mediator levels but also cells at local organ sites, which must not be neglected. Intracellular cytokine detection using flow cytometry overcomes these problems by allowing reliable information regarding the cellular source of cytokines on a single cell level to be gained. However, the method requires a time demanding work-flow including *ex vivo *stimulation, antibody staining, and flow cytometric analysis. This is obviously the reason why the method has not found great clinical acceptance over the years. Here, we regard it as a relevant method to help critical care practitioners ascertain how the inflammatory response might be altered with time.

## Conclusions

As our data were derived from very narrow intervals of measurements, they might contribute to a more detailed understanding of the early immunoalterations recognized after severe trauma. It can be concluded that, as previously postulated, an immediate hyperactivation of circulating monocytes is rapidly followed by a substantial paralysis of cell function. Moreover, our findings clearly demonstrate that the restricted capacity of monocytes to produce proinflammatory cytokines after severe injury is not only an *in vitro *phenomenon but also undistinguishable associated with the onset of organ dysfunction in the clinical scenario.

## Key messages

• We found a significant reduction of LPS reactivity in circulating monocytes staining positive for TNFα, IL-1β, IL-6, and IL-8 between 12 and 48 hours after injury.

• In particular, 24 hours after trauma TNFα *de novo *synthesis was strongest blunted after LPS challenge followed by IL-6, IL-1β, and finally IL-8, which was also reduced but not as much as the previous mentioned mediators.

• In the further time course, all analyzed cytokines showed a rapid and nearly complete recovery towards normal.

• The present study might complement the current picture of posttraumatic cytokine dynamics and suggest that monocytic capacity recovers up to 72 hours post trauma followed by a second decrease up to day 10 post injury.

## Abbreviations

ELISA: enzyme-linked immunosorbent assay; FITC: fluoresceinisothiocyanate; IL: interleukin; LPS: lipopolysaccharide; MFI: mean fluorescence intensity; MODS: multiple organ dysfunction syndrome; MOF: multiple organ failure; NaN_3_: sodium azide; NISS: New Injury Severity Score; PBS: phosphate buffered saline; PE: phycoerythrin; PE-Cy5: phycoerythrin-cyanin-5; TNF: tumor necrosis factor.

## Competing interests

The authors declare that they have no competing interests.

## Authors' contributions

CK, SZ, and PB contributed to study design, data collection and analysis, and drafted the manuscript. WEM, EF, and MJ contributed to study design and manuscript review.
